# Mechanisms Underpinning Increased Plasma Creatinine Levels in Patients Receiving Vemurafenib for Advanced Melanoma

**DOI:** 10.1371/journal.pone.0149873

**Published:** 2016-03-01

**Authors:** Charlotte Hurabielle, Evangéline Pillebout, Thomas Stehlé, Cécile Pagès, Jennifer Roux, Pierre Schneider, Sylvie Chevret, Cendrine Chaffaut, Anne Boutten, Samia Mourah, Nicole Basset-Seguin, Emmanuelle Vidal-Petiot, Céleste Lebbé, Martin Flamant

**Affiliations:** 1 Dermatology Department, Hôpital Saint-Louis, Assistance-Publique-Hôpitaux de Paris, Paris, France; 2 Nephrology Department, Hôpital Saint-Louis, Assistance-Publique-Hôpitaux de Paris, Paris, France; 3 Physiology Department, DHU Fire, Hôpital Bichat, Assistance-Publique-Hôpitaux de Paris, Paris, France; 4 Biostatistic Department, Hôpital Saint-Louis, Assistance-Publique-Hôpitaux de Paris, ECSTRA Team, Inserm UMR-1153, Université Paris 7 Diderot, Paris, France; 5 Biochemistry Department, Hôpital Bichat, Assistance-Publique-Hôpitaux de Paris, Paris, France; 6 Pharmacology and Genetic Department, Hôpital Saint-Louis, Assistance-Publique-Hôpitaux de Paris, Paris, France; 7 Inserm Unit 976, Hôpital Saint-Louis, Université Paris Diderot, Sorbonne Paris Cité, Paris, France; 8 Université Paris Diderot, Sorbonne Paris Cité, Paris, France; University of Sao Paulo Medical School, BRAZIL

## Abstract

**Context:**

Serum creatinine has been reported to increase in patients receiving Vemurafenib, yet neither the prevalence nor the mechanism of this adverse event are known.

**Objective:**

We aimed to evaluate the frequency and the mechanisms of increases in plasma creatinine level in patients receiving Vemurafenib for advanced melanoma.

**Methods:**

We performed a retrospective monocentric study including consecutive patients treated with Vemurafenib for an advanced melanoma. We collected clinical and biological data concerning renal function before introduction of Vemurafenib and in the course of monthly follow-up visits from March 2013 to December 2014. Cystatin C-derived glomerular filtration rate was evaluated before and after Vemurafenib initiation, as increase in serum cystatin C is specific to a decrease in the glomerular filtration rate. We also performed thorough renal explorations in 3 patients, with measurement of tubular secretion of creatinine before and after Vemurafenib initiation and a renal biopsy in 2 patients.

**Results:**

70 patients were included: 97% of them displayed an immediate, and thereafter stable, increase in creatinine (+22.8%) after Vemurafenib initiation. In 44/52 patients in whom Vemurafenib was discontinued, creatinine levels returned to baseline. Serum cystatin C increased, although proportionally less than serum creatinine, showing that creatinine increase under vemurafenib was indeed partly due to a renal function impairment. In addition, renal explorations demonstrated that Vemurafenib induced an inhibition of creatinine tubular secretion.

**Conclusion:**

Thus, Vemurafenib induces a dual mechanism of increase in plasma creatinine with both an inhibition of creatinine tubular secretion and slight renal function impairment. However, this side effect is mostly reversible when Vemurafenib is discontinued, and should not lead physicians to discontinue the treatment if it is effective.

## Introduction

Vemurafenib is an oral BRAF inhibitor which has been shown to increase progression-free and overall survival in patients with advanced melanoma [1]. Like other researchers, we have observed an increase in serum creatinine levels in patients receiving Vemurafenib, yet neither the prevalence nor the mechanism underpinning this adverse event are known [2–4].

Indeed, an increase in serum creatinine under treatment can have numerous causes, sometimes intricate. It can be the consequence of

- altered intraglomerular hemodynamics, as with nonsteroidal anti-inflammatory drugs (NSAIDs) or angiotensin-converting enzyme (ACE) inhibitors, for example,
- tubular necrosis by direct tubular cell toxicity (aminoglycosides or amphotericin B) or via rhabdomyolisis (statins) or crystal nephropathy (foscarnet or indinavir)
- inflammation of the interstitium, which can result from an allergic response (acyclovir, NSAIDs or indinavir), or non-inflammatory glomerular lesions (gold therapy or NSAIDs),
- a thrombotic microangiopathy (clopidogrel, quinine or anti-VEGF) [5].

As with NSAIDs, a single drug can induce renal toxicity via several different mechanisms.

Another mechanism leading to elevated serum creatinine in this context could be an inhibition of the normal tubular secretion of creatinine. This can occur when drugs interfere with protein carriers in the proximal tubule as demonstrated for Cimetidine, Trimethoprim, Pyrimethamine and Salicylates [6].

We aimed to evaluate the frequency and the mechanisms of increases in serum creatinine levels in patients receiving Vemurafenib for advanced melanoma.

## Materials and Methods

**Fig 1:**
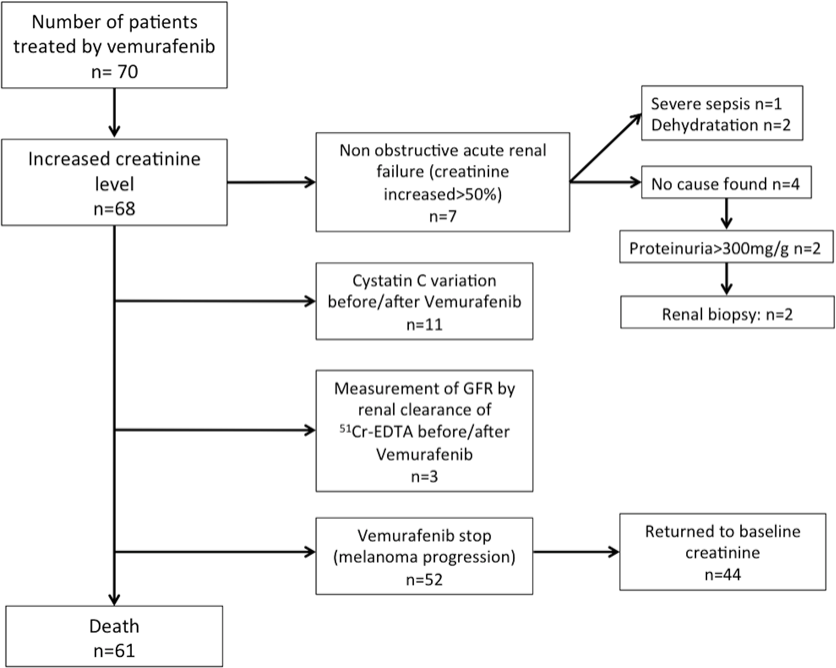
Flow-chart of patients included.

We designed a retrospective, monocentric study including all patients treated with Vemurafenib for an advanced melanoma from March 2013 to December 2014 (Fig 1). We collected clinical and biological data concerning renal function before introduction of Vemurafenib and at monthly follow-up visits for all the patients. Glomerular filtration rate (GFR) was estimated with the MDRD (Modification of Diet In Renal Disease) formula, from serum creatinine concentration value measured before introducing Vemurafenib. The local ethics committees (Comité de Protection des Personnes CPP XI from Saint-Germain-en-Laye) approved the study and all the subjects gave written informed consent.

In order to better define the mechanisms underlying elevation in serum creatinine levels under Vemurafenib, in 11 patients, the change in GFR after Vemurafenib initiation was assessed from cystatin C variation, and was compared with that derived from serum creatinine variation. Indeed, cystatin C is a low-molecular-weight protease inhibitor produced by all nucleated cells, freely filtered by the glomerulus and not secreted by the tubules. Its concentration is inversely correlated with GFR: when cystatin C increases in the blood, it provides a reliable indication of the decrease in the glomerular filtration rate, that is to say an impairment in renal function. In other words, the decrease of 1/Cys reflects the actual decrease in the GFR [7]. Conversely, when plasma creatinine increases, it suggests a real impairment of renal function, and/or an inhibition of tubular secretion of creatinine.

We further explored the mechanisms underpinning creatinine variations under Vemurafenib therapy in 3 patients by performing gold standard measurements of GFR and of tubular handling of creatinine before and after initiation of Vemurafenib.

Data were recorded during a 5-hour in-person visit to our physiology department. Measured GFR (mGFR) was determined by a renal clearance of 51Cr-EDTA as previously described [8]. Briefly, after a single intravenous bolus injection of 1.8 to 3.5 MBq of 51Cr-EDTA (GE Healthcare, Velizy, France), we allowed 1.5h for distribution of the tracer in the extracellular fluid, and renal 51Cr-EDTA clearance was determined from the average of 6 consecutive 30-min clearance periods, considered as mini-equilibrium periods. A blood sample was drawn for measurement of plasma concentration of the tracer in the middle of each period. For each period, mGFR was calculated as the urinary output of 51Cr-EDTA (urinary concentration of 51Cr-EDTA x urinary output) divided by the corresponding plasma concentration of the tracer. mGFR was the average of the values obtained for the 6 periods. The same urine samples were used to determine clearance of creatinine. The proportion of creatinine clearance due to its tubular secretion was calculated as the average of creatinine clearance–mGFR for each 30min period.

## Results

70 patients were included in the study (30 males, median age at diagnosis 56.5 years, interquartile range 47.3–69.0, median follow-up 5 months). Detailed patient characteristics are shown in Table 1.

**Table 1:** Main clinical and biological data of the 70 patients at baseline and during the treatment.

Age, mean (interquartile range)	56.5 years (47.3–69.0 years)
Men (n) / Women (n)	30 / 40
Weight	70 kg
Vemurafenib dosage at initiation (n)	1920mg/day (n = 70)
Median duration of the treatment	5 months
Creatinine before vemurafenib initiation, mean (standard deviation)	78 μmol/L (24.7)
Creatinine one month after vemurafenib initiation, mean (standard deviation)	105 μmol/L (42.6)
Median variation between creatinine at 1 month versus at initiation (%)	+22.8%
Creatinine after vemurafenib stop, (n), mean (standard deviation)	n = 52, 76.3 μmol/L (22.2)

At the first visit after Vemurafenib initiation (one month after first administration of the treatment), a significant increase in serum creatinine levels (>5%) occurred in 68/70 patients (97%), with a median variation of 22.8%. After this initial increase, creatinine remained stable. In 44 out of 52 patients in whom Vemurafenib was discontinued (mostly because of progression of the melanoma), serum creatinine levels (measured within two months after treatment cessation) returned to baseline. All the patients underwent routine urine test strip performed routinely, and if it was positive for any parameter, cytobacteriological urine tests were performed and the protein to creatinine ratio was measured in a urine sample. After excluding 8 patients who had urinary tract infections, we analyzed 30 patients for whom we obtained urine samples. It can be noted that for these 30 patients, we found:

- no proteinuria in 14 (47%), defined by a protein to creatinine ratio <300mg/g
- mild proteinuria, ranging from 324 to 1636 mg/g (median 488.9mg/g), in 16/30 patients (53%),
- no significant presence of red or white blood cells.

Non-obstructive acute renal failure (serum creatinine level increase >50%) occurred in 7 patients, within 1 month of treatment initiation and was attributed to a severe sepsis in 1 patient and to dehydration in 2 other patients. In 4 patients, no causal factor was identified. In 2 of these 4 patients, serum creatinine concentration was multipled by 2.2 and 2.0, respectively, with a protein to creatinine ratio >500mg/g. Renal biopsies were therefore performed, and showed relatively well-preserved renal parenchyma without clearly visible injury apart from toxic tubular epithelial cells injury with tubular proteic cylinders and proteic clusters in the cytoplasm of proximal tubular epithelial cells. There was no glomerular or vascular lesion. Immunofluorescence was normal. Electronic microscopy showed a vacuolization of the proximal tubule epithelium with the presence of cytoplasmic debris in the tubular lumen for the first patient, and for the second a slight atrophy of the proximal and distal convoluted tubules.

For the 2 remaining patients, renal biopsy was contraindicated because they had only one kidney (congenital solitary kidney and nephrectomy for severe renal dysplasia respectively).

In all seven patients, serum creatinine levels returned to baseline level within 1 month after Vemurafenib cessation.

The almost systematic, moderate, immediate and thereafter stable, increase in creatinine, most often reversible, was highly suggestive of a mechanism at least in part independent from a decrease in GFR, related to an inhibition of the tubular secretion of creatinine. We performed further studies to determine the mechanism underpinning the increase in serum creatinine levels observed.

In 11 patients, the decrease in GFR after Vemurafenib initiation, assessed from the variation of 1/cystatin, was 9.4%, and that derived from the variation of 1/serum creatinine was 16.7% (p = 0.06). The decrease in the 1/cystatin ratio meant that there was a median 9.4% decrease in GFR under Vemurafenib. However, the GFR was proportionally lower when estimated from serum creatinine than when derived from serum cystatin C. This meant that there was an additional mechanism explaining the increase in serum creatinine. This could be an inhibition of the normal tubular secretion of creatinine, which thus could induce an elevation of serum creatinine.

**Fig 2:**
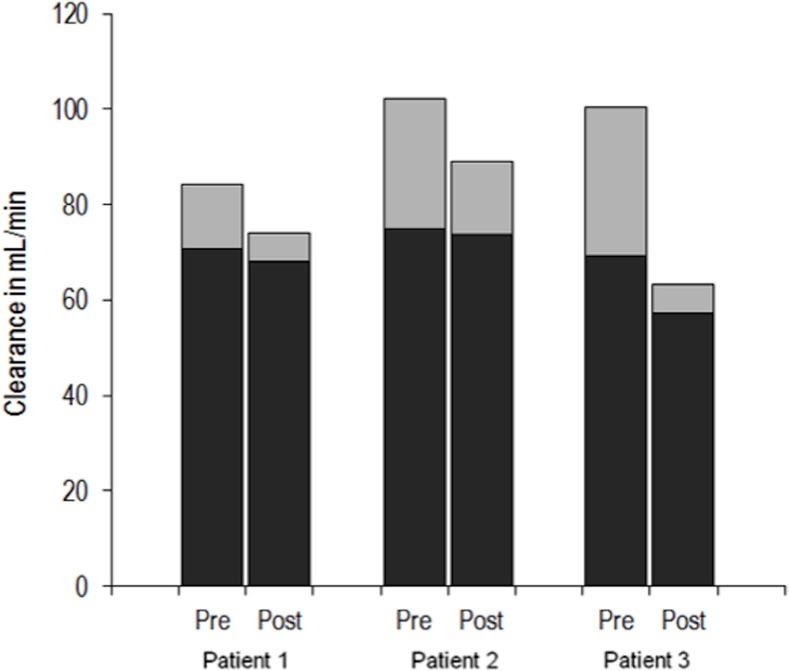
Renal handling of creatinine before and after introduction of Vemurafenib. For each patient and each visit, the black part of the bar represents GFR (urinary clearance of ^51^CrEDTA) and the grey part of the bar represents the proportion of creatinine clearance due to secretion, the whole bar representing overall urinary clearance of creatinine. For each patient, the proportion of clearance of creatinine due to its secretion decreased under Vemurafinib treatment by 55, 43 and 81% for patients 1, 2 and 3 respectively.

To test the hypothesis that the increase in serum creatinine levels could result from a decrease in tubular secretion of creatinine, we performed thorough renal explorations, including precise measurement of the proportion of creatinine clearance due to tubular secretion and of GFR in three patients pre- and post Vemurafenib initiation. As shown in Fig 2 and Table 1, tubular secretion of creatinine decreased under treatment in all 3 patients, by 55, 43 and 81% respectively. Conversely, mGFR was stable in the first two patients and decreased in the third patient only (83.3, 88.2 and 65.4 vs 81.7, 89.7 and 56.6 ml/min/1.73m^2^, before and after Vemurafenib initiation, respectively). It can be noted that all three patients developed moderate signs of proximal tubular lesions under Vemurafenib therapy namely non-glomerular proteinuria, aminoaciduria and an increased fractional excretion of uric acid (Table 2). No patients displayed phosphate or glucose loss (data not shown).

**Table 2:** Renal function before and after introduction of Vemurafenib.

		sCr mg/dL	mGFR ML/min	CCr mL/min	CCrS mL/min	PCR Mg/g	Uric Acid FE %	Aminoaciduria Y/N
	Pre	0.63	70.8	89.5	13.5	13.9	3.8	N
Patient 1	Post	0.76	68.0	74.0	6.0	723.1	7.2	Y
	Variation	21%	-4%	-17%	-55%			
	Pre	0.60	74.9	102.1	27.2	181.2	7.0	N
Patient 2	Post	0.63	73.7	89.2	15.5	884.0	14.0	Y
	Variation	5%	-2%	-13%	-43%			
	Pre	0.86	69.2	100.3	31.1	135.3	9.8	ND
Patient 3	Post	1.15	57.3	63.2	6.0	319.1	13.0	Y
	Variation	34%	-17%	-37%	-81%			

BSA: Body Surface Area; sCr: serum creatinine concentration; mGFR: measured glomerular filtration rate; CCr: clearance of creatinine; CCr-S: part of CCr due to secretion (calculated as CCr-mGFR); PCR: Protein Creatinine Ratio; FE: fractional excretion; ND: not done.

## Discussion

Creatinine is freely filtered in the glomerulus, and not reabsorbed or metabolized by the tubule, which makes it a near-ideal endogenous GFR marker. However, significant tubular secretion of creatinine occurs in the proximal tubule. The proportion of creatinine clearance resulting from its tubular secretion is approximately 20–30% but varies inter-individually [9]. As creatinine clearance equals GFR plus tubular secretion, an acute increase in serum creatinine can reflect a GFR decline and/or an inhibition of the tubular secretion [6]. Inhibition of this sort occurs when a drug interferes with basocellular/apical transporters responsible for the transcellular tubular transport of creatinine. This phenomenon was first described with Cotrimoxazole and Cimetidine [10,11]. In strong support of our demonstration of a decrease in tubular secretion of creatinine, Vemurafenib has been shown to be carried by OCT2, which is responsible for the basocellular uptake of creatinine in the proximal tubule. This explains why some patients have an increase in serum creatinine with no modification in GFR: thus, Vemurafenib inhibits the tubular secretion of creatinine in these patients, which increases serum creatinine level without any renal function impairment.

However, true GFR decline and/or signs of proximal tubule lesions do occur in some patients, in addition to the inhibition of the tubular secretion. Indeed, Vemurafenib induced proximal tubular epithelial cell injury in the 2 cases for whom we obtained renal biopsy, and led to mild proteinuria in 19/70 patients (27%). Further larger studies including biomarkers of tubular toxicity are required to establish to prevalence of tubular dysfunction under Vemurafenib therapy. In clinical practice, renal function and signs of proximal tubule dysfunction should be carefully monitored after Vemurafenib initiation.

**Fig 3:**
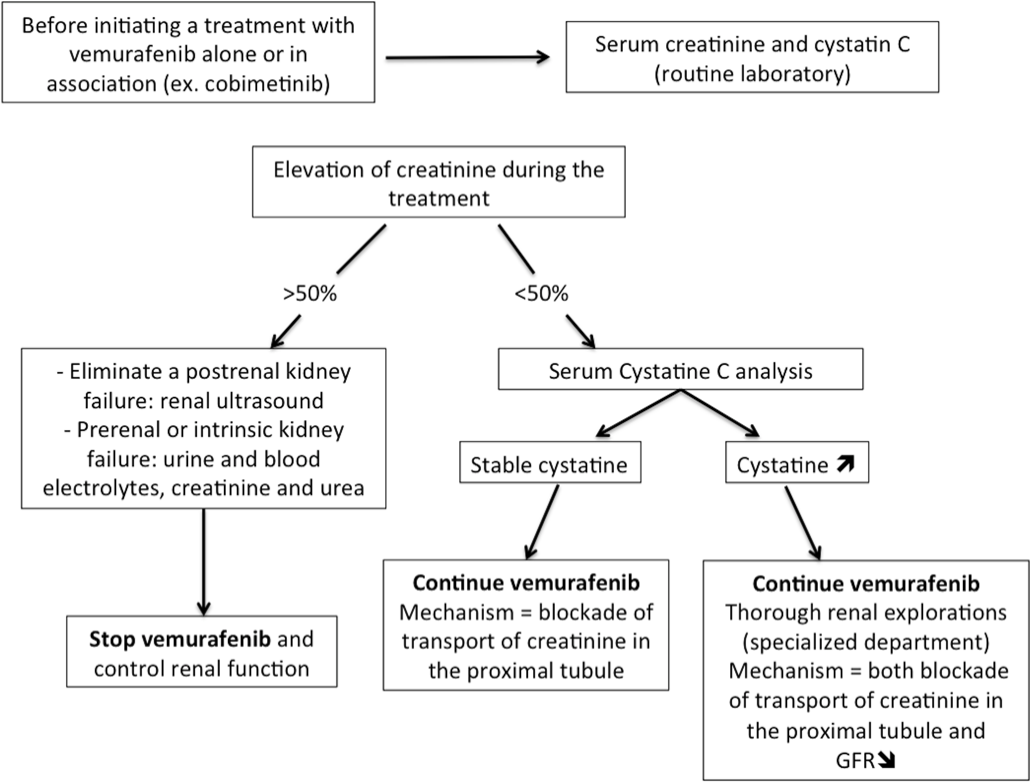
Decision-tree to manage elevation of creatinine under Vemurafenib.

In conclusion, Vemurafenib induces an immediate, stable and usually reversible increase in serum creatinine. An inhibition of the tubular secretion of creatinine occurs under Vemurafenib therapy and is one feature of a proximal tubulopathy, which may or may not be associated with a decrease in GFR. Thus, the interpretation of creatinine variations in patients treated with Vemurafenib is difficult, as they may reflect both a blockade of transport of creatinine in the proximal tubule and GFR modifications. Currently, the standard of care in BRAF-mutated advanced melanoma is a combination of BRAF and MEK inhibitors [12,13]. Therefore, in patients treated with Vemurafenib and Cobimetinib or Vemurafenib alone, we propose a decision tree to help clinicians manage the elevation of creatinine under these treatments (Fig 3). Thus, we propose a test for serum creatinine and cystatin C before the beginning of the treatment and in the course of monthly follow-up thereafter.

Finally, the data from this study is reassuring: apart from rare cases of DRESS induced by Vemurafenib and cases of severe acute renal failure, an increase in sCr below 50% and/or moderate signs of tubular dysfunction should not lead clinicians to discontinue the treatment if it is effective.

## Supporting Information

S1 Table Protein creatinine ratio in patients having positive urine test strip without urinary tract infection. (DOCX)
