# Supplementary material for: Mechanisms Underpinning Increased Plasma Creatinine Levels in Patients Receiving Vemurafenib for Advanced Melanoma
Source: PLoS One. 2016 Mar 1;11(3):e0149873. doi: 10.1371/journal.pone.0149873 (PMC4773169; doi:10.1371/journal.pone.0149873)
Supplement: S1 Table — (DOCX) [file pone.0149873.s001.docx]

| PATIENT n° | urine proteine creatinine ratio mg/g | >300 | white cells | red cells |
| --- | --- | --- | --- | --- |
| 1 | 324 | 1 | 0 | 0 |
| 2 | 270 | 0 | 0 | 0 |
| 3 | 36 | 0 | 0 | 0 |
| 4 | 90 | 0 | 0 | 0 |
| 5 | 780,6629834 | 1 | 0 | 0 |
| 6 | 1800 | 1 | 0 | 0 |
| 7 | 173,0769231 | 0 | 0 | 0 |
| 8 | 165,7894737 | 0 | 0 | 0 |
| 9 | 476,4705882 | 1 | 0 | 0 |
| 10 | 535,4237288 | 1 | 0 | 0 |
| 11 | 236,8421053 | 0 | 0 | 0 |
| 12 | 370 | 1 | 0 | 0 |
| 13 | 324 | 1 | 0 | 0 |
| 14 | 1575 | 1 | 0 | 0 |
| 15 | 105,8823529 | 0 | 0 | 0 |
| 16 | 94,73684211 | 0 | 0 | 0 |
| 17 | 355,5555556 | 1 | 0 | 0 |
| 18 | 411,1111111 | 1 | 0 | 0 |
| 19 | 253,6912752 | 0 | 0 | 0 |
| 20 | 241,0714286 | 0 | 0 | 0 |
| 21 | 60 | 0 | 0 | 0 |
| 22 | 514,2857143 | 1 | 0 | 0 |
| 23 | 207,6923077 | 0 | 0 | 0 |
| 24 | 450 | 1 | 0 | 0 |
| 25 | 1356,338028 | 1 | 0 | 0 |
| 26 | 189 | 0 | 0 | 0 |
| 27 | 434,8314607 | 1 | 0 | 0 |
| 28 | 1635,693878 | 1 | 0 | 0 |
| 29 | 501,0810811 | 1 | 0 | 0 |
| 30 | 189 | 0 | 0 | 0 |
